# Intraspecific Diversity of Microbial Anti-Inflammatory Molecule (MAM) from *Faecalibacterium prausnitzii*

**DOI:** 10.3390/ijms23031705

**Published:** 2022-02-01

**Authors:** Sandrine Auger, Camille Kropp, Esther Borras-Nogues, Wasaporn Chanput, Gwenaelle Andre-Leroux, Oscar Gitton-Quent, Leandro Benevides, Natalia Breyner, Vasco Azevedo, Philippe Langella, Jean-Marc Chatel

**Affiliations:** 1 Université Paris-Saclay, INRAE, AgroParisTech, Micalis, 78352 Jouy-en-Josas, France; sandrine.auger@inrae.fr (S.A.); camille.kropp@inrae.fr (C.K.); esther.borras-nogues@inrae.fr (E.B.-N.); fagiwpc@ku.ac.th (W.C.); oscar.gitton-quent@inrae.fr (O.G.-Q.); ljbenevides@gmail.com (L.B.); natalia.martinsbreyner@gmail.com (N.B.); philippe.langella@inrae.fr (P.L.); 2 Maiage, INRAE, 78350 Jouy-en-Josas, France; gwenaelle.andre-leroux@inrae.fr; 3 Institute of Biological Sciences, Federal University of Minas Gerais, Belo Horizonte 31270-901, MG, Brazil; vascoariston@gmail.com

**Keywords:** *Faecalibacterium prausnitzii*, microbial anti-inflammatory molecule, probiotic

## Abstract

The commensal bacterium *Faecalibacterium prausnitzii* has unique anti-inflammatory properties, at least some of which have been attributed to its production of MAM, the Microbial Anti-inflammatory Molecule. Previous phylogenetic studies of *F. prausnitzii* strains have revealed the existence of various phylogroups. In this work, we address the question of whether MAMs from different phylogroups display distinct anti-inflammatory properties. We first performed wide-scale identification, classification, and phylogenetic analysis of MAM-like proteins encoded in different genomes of *F. prausnitzii*. When combined with a gene context analysis, this approach distinguished at least 10 distinct clusters of MAMs, providing evidence for functional diversity within this protein. We then selected 11 MAMs from various clusters and evaluated their anti-inflammatory capacities in vitro. A wide range of anti-inflammatory activity was detected. MAM from the M21/2 strain had the highest inhibitory effect (96% inhibition), while MAM from reference strain A2-165 demonstrated only 56% inhibition, and MAM from strain CNCM4541 was almost inactive. These results were confirmed in vivo in murine models of acute and chronic colitis. This study provides insights into the family of MAM proteins and generates clues regarding the choice of *F. prausnitzii* strains as probiotics for use in targeting chronic inflammatory diseases.

## 1. Introduction

*Faecalibacterium prausnitzii* has been consistently reported to contribute to a healthy gut immune response due to its anti-inflammatory properties [1,2]. A decrease in the abundance of *F. prausnitzii* is associated with the occurrence of various gastrointestinal disorders, such as inflammatory bowel diseases, irritable bowel syndrome, colorectal cancer, and obesity [3,4]. The anti-inflammatory activity of *F. prausnitzii* has been linked to its ability to produce butyrate, which serves as the preferred energy source of colonic bacteria and has been found to influence the differentiation of T helper 17 cells (Th17) and regulatory T cells (Treg), the development of extracellular polymeric matrix, and the creation of peptides derived from the Microbial Anti-inflammatory Molecule (MAM) [1,5,6,7]. The MAM protein, which is only produced by *F. prausnitzii*, also appears to play a role in the unique anti-inflammatory properties of this bacterium. Several in vitro and in vivo assays in animal models have revealed that MAM is able to block the NF-kB pathway and consequently reduce production of the IL-8 cytokine [1,8]. In two different mouse models of chemically induced inflammation (dinitrobenzene sulfonic acid (DNBS) and dextran sulfate sodium (DSS), respectively), MAM produced by cells of the host mucosa (following cDNA delivery) showed the ability to reduce the Th1, Th2, and Th17 immune responses [1,8]. Moreover, in both models, MAM from the A2-165 strain was able to increase levels of the IL-10 and TGFβ cytokines, which are involved in the process of Treg differentiation that is responsible for intestinal homeostasis [8]. For this reason, *F. prausnitzii* currently represents one of the most promising taxa for the development of next-generation probiotics (NGPs) [9,10,11].

Research by Martin et al. (2017) highlighted the fact that different strains of *F. prausnitzii* demonstrate different anti-inflammatory properties [12,13]. Specifically, these authors evaluated the immune-modulating properties of *F. prausnitzii* in vitro by characterizing its ability to decrease IL-8 production in TNFα-induced HT-29 cells and to stimulate IL-10 production in PBMC culture. They found that all 13 tested strains of *F. prausnitzii* tended to decrease IL-8 secretion, but not to the same extent. Instead, only four strains—A2-165, CNCM4543, CNCM4573, and L2-6—were able to induce IL-10 production. The two most promising strains were A2-165 and CNCM4543, which are closely related at the genetic level [13].

The phylogenetic relationships among strains of *F. prausnitzii* are still under debate. Previous work based on 16S rRNA analysis of isolates suggested the presence of two phylotypes [14,15]. More recently, our group performed whole-genome comparisons of 17 genomes [13]. Thirteen of these clustered into three distinct phylogroups—A, B, and C—while the remaining four strains (L2-6, CNCM4575, AHMP21, and CNCM4541) were assigned to separate clusters. Subsequently, Fitzgerald et al. proposed separating strains of *F. prausnitzii* into two new species, *F. prausnitzii sensu stricto* and *F. moorei* [16], and in 2021, two new species of *Faecalibacterium*, named *F. butyricigenerans* and *F. longum*, were described [17]. Analyses of *Faecalibacterium*-like metagenome-assembled genomes, which were reconstructed from human gut metagenomes, highlighted the high level of diversity existing within this genus [18]. The 12 different species-level clades identified may have different functions, with potentially diverse contributions to health or disease status.

In the present study, we addressed the question of whether MAM proteins from different phylogroups might contribute to the dissimilarities in anti-inflammatory properties among strains. We first focused on the identification, classification, and analysis of MAM-like proteins from different strains. The use of a sequence similarity network and gene context analysis allowed us to elucidate the relationships between them, which enabled us to classify the identified MAMs into at least 10 distinct families. We then, for the first time, characterized several representative MAMs from different phylogroups with respect to their anti-inflammatory effects in vitro and in vivo in a mouse model of DNBS-induced inflammation. We report here that these MAMs present a wide range of anti-inflammatory activity, which should be taken into account in the selection of strains for use as NGPs.

## 2. Results

### 2.1. Gene Context Analysis

**Figure 1:**
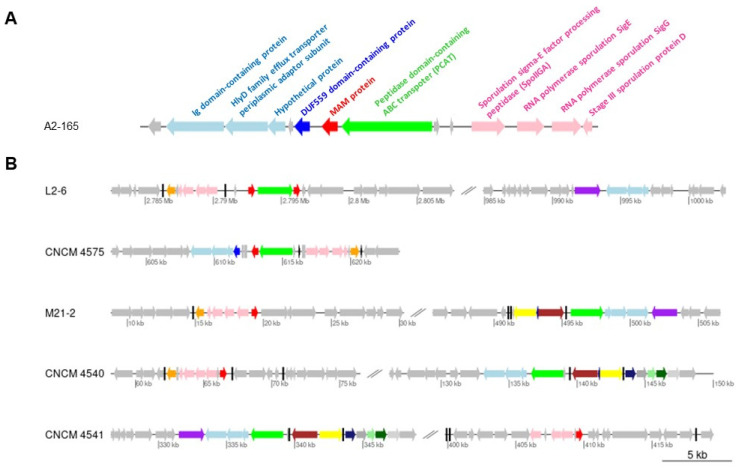
Genetic organization of the nucleotide region encoding the MAM protein in *F. prausnitzii*. Arrows indicate the direction of gene transcription. (**A**) Organization in the A2-165 strain. The putative functions of the genes are indicated. (**B**) Comparison of the organization in several representative strains of *F. prausnitzii*. Genes conserved in different genomes are indicated by the same color label.

We first explored the genetic contexts of the different MAM-encoding genes, named here *mam*, within representative strains of *F. prausnitzii* from different phylogroups (Figure 1). In the genome of the A2-165 strain, *mam* is flanked by an upstream gene that encodes a protein with a DUF559 domain and a downstream gene that encodes a peptidase-containing ABC transporter (PCAT) (Figure 1A). The upstream region also includes a cluster of open reading frames (ORFs) encoding three hypothetical proteins. To identify conserved domains within these protein sequences, we queried the Conserved Domain Search Service [19] and found that two of these sequences demonstrated affinity with, respectively, an Ig domain-containing protein and an HlyD family efflux transporter periplasmic adaptor subunit (Figure 1A). In addition, in the vicinity of *mam* there were also four ORFs putatively involved in the sporulation process.

When we compared the chromosomal regions carrying the *mam* gene among different strains, we identified variations in their genetic arrangements relative to *mam* (Figure 1B). For example, the L2-6 genome contains two *mam* genes, which are located on either side of the PCAT gene. In the A2-165, L2-6, and CNCM4575 strains, the *mam* genes are close to the PCAT gene, while in the other genomes PCAT is located in another chromosomal region. Remarkably, the proximity of the PCAT-encoding gene to the cluster of three ORFs encoding an efflux transporter periplasmic adaptor is conserved in many *F. prausnitzii* genomes (Figure 1B), which suggests their mutual involvement in a common transport function. Finally, it is notable that the sporulation cluster is also located near the *mam* gene in all *F. prausnitzii* genomes.

### 2.2. Analysis of the Promoter Regions

**Figure 2:**
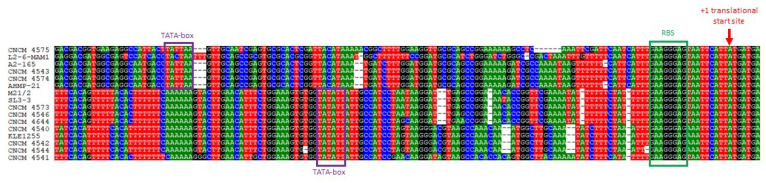
Sequence alignment of promoter regions from *mam* genes. We examined 200 nucleotides upstream of the annotated translational start site, indicated by +1. The sequences of the ribosome binding site are indicated by a green frame. The sequences of the TATA box are indicated by purple frames.

In order to assess whether expression of the *mam* genes could be conserved among strains from different phylogroups, we compared their promoter regions by sequence alignment (Figure 2). This revealed high levels of sequence conservation from nucleotide −20 to nucleotide −1 upstream of the translation start codon. These regions contain a Shine–Dalgarno motif that probably acts as a ribosomal binding site (RBS). By contrast, we observed strong divergence in the sequences upstream of the RBS. From recently published RNAseq data [20], we determined that transcription of the *mam* gene begins 110-bp upstream of its translational start site. This region contains a TATA box, where formation of the transcription pre-initiation complex takes place (Figure 2). Within the sequence alignment, we could distinguish at least two main types of promoters depending on the location of the putative TATA box: (i) short promoters, in which the TATA box was about 80 nucleotides from the translation start codon; and (ii) large promoters, in which there was a TATA box-like motif about 105 nucleotides from the translation start codon. This strongly suggests that transcriptional regulation of the MAM-encoding genes is not conserved among *F. prausnitzii* strains.

### 2.3. Cluster Analysis of the MAM Proteins

Strains of *F. prausnitzii* can be classified into at least eight phylogroups [13,16]. Here, we investigated the relationships among MAM-like proteins to determine if they reflected larger patterns among the strains. We first built a dataset using Psi-BLAST searches (Table S2). The sequences from the MAM dataset were compared all-against-all using BLAST in order to find clusters of similar sequences and visualize the sequence relationships, as implemented in the CLuster ANalysis of Sequences (CLANS) program [21].

**Figure 3:**
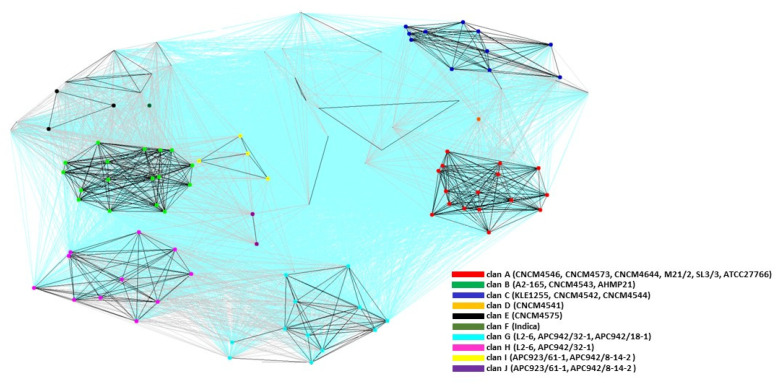
Cluster map of identified full-length MAM proteins. The dataset of 108 MAM proteins was clustered using CLANS, which classified all MAM proteins into families. CLANS runs BLAST on a given set of sequences and clusters them in 3D according to their all-against-all pairwise similarity. A 2D representation was obtained by seeding sequences randomly in the arbitrary distance space. For each clan, some known member strains are indicated in parentheses in the legend. Connections with a *P*-value higher than 1E-22 are rendered in blue, those with a P-value between 1E-22 and 1E-33 are rendered in gray, and those with a *P*-value lower than 1E-33 are rendered in black.

CLANS identified at least 10 families (clans) of MAM proteins (Figure 3). Some groups contained only a few sequences, which was probably the result of under-representation of the corresponding strains in the databases used. Based on their evolutionary descent, the phylogeny of MAMs broadly overlapped that of the phylogroups, in that three clans—A, B, and C—included MAMs from strains in phylogroups A, B, and C, respectively. However, certain inconsistencies were noted. For example, the MAM from strain AHMP21 was detected within clan B while this strain was reported to belong to a separate phylogroup [13,16]. Conversely, strain Indica is thought to belong to phylogroup B, but its MAM is clustered in its own distinct clan. The MAM from strain CNCM4541 constituted a separate clan, reflecting the separate phylogroup of that strain. Interestingly, in the strain that contained two *mam* genes located in the same genomic neighborhood (the L2-6 strain; Figure 1), the MAM proteins were assigned to separate, well-defined clans (clans G and H), but the relative positions of these clans indicated a shared evolutionary history. The same pattern was observed for strains APC923/61-1 and APC942/8-14-2, whose two MAMs clustered in clans I and J, respectively. This suggests that in strains with two *mam* genes, duplication of the *mam* gene is followed by differentiation. This prompted us to perform further phylogenetic analyses of the MAM family to gain insight into the evolutionary links among these proteins.

### 2.4. Phylogenetic Analysis of the MAM Protein Family

**Figure 4:**
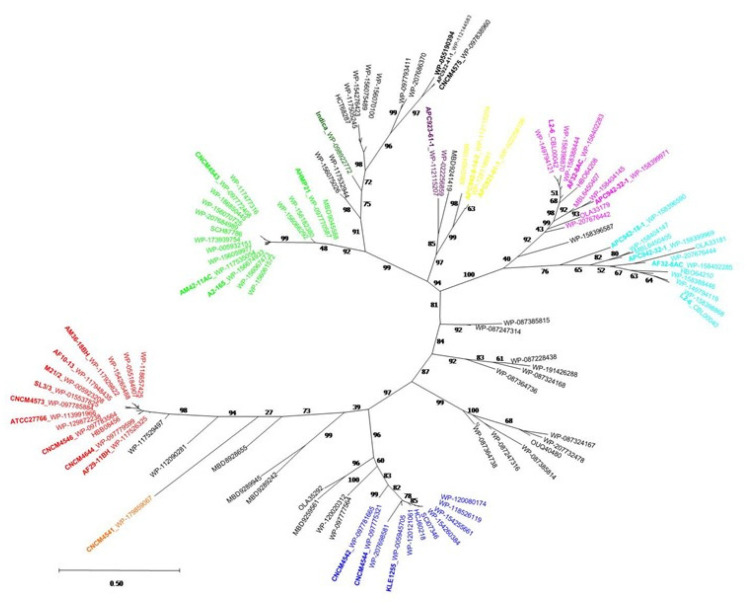
Unrooted maximum likelihood phylogenetic tree of MAM proteins. The MAM phylogeny was reconstructed using MEGA-X (Materials and Methods). Bootstrap support values are shown.

The dataset of MAM proteins was used to construct phylogenetic trees (Materials and Methods), which were built from multiple-sequence alignments using the Maximum Likelihood (ML) method. The resulting ML tree illustrated the evolutionary diversity of MAM proteins (Figure 4) and a clear separation of MAM proteins into several distinct lineages (bootstrap > 95). These results were consistent with those obtained with CLANS (Figure 3). The MAM sequence from the Indica strain forms a separate lineage, whose closest evolutionary neighbors are lineages B and E. With respect to the strains containing two *mam* genes, the MAMs from L2-6 and APC942/32-1 form two separate clades, G and H, originating from the same branch (bootstrap = 92). Likewise, the MAM proteins from strains APC923/61-1 and APC942/8-14-2 also form two clades, I and J (bootstrap = 97), which diverge from the same branch.

### 2.5. In Vitro Anti-Inflammatory Activity of MAMs from Different Clans

**Figure 5:**
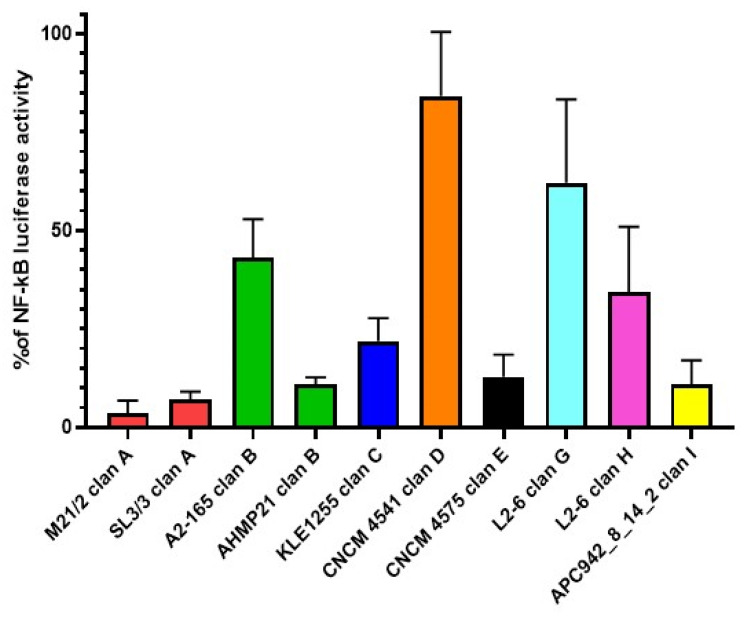
In vitro anti-inflammatory properties of MAM proteins. Anti-inflammatory properties of the MAM proteins were evaluated using an NF-kB reporter luciferase assay. The HEK 293 cell line was transfected with an NF-kB luciferase plasmid, a pCarma1 plasmid, and a probiH1 plasmid containing MAM cDNA from different strains of *F. prausnitzii*. Results are expressed as % of NF-kB luciferase activity, with 100% representing the value obtained after transfection with NF-kB luciferase, Carma1, and proBiH1 EMPTY plasmids. Each plasmid was tested in triplicates (technical replicate) and experiments were performed three times (biological replicate). Results are expressed as mean of the three biological replicates.

We performed multiple alignments of the MAM sequences from the different clans (Figure S1) and selected several representative MAM proteins belonging to different strains (Table S1): M21/2 and SL3/3 from clan A; A2-165 and AHMP21 from clan B; KLE1255 from clan C; CNCM4541 from clan D; CNCM4575 from clan E; L2/6 from clan G and clan H; and APC942/8-14-2 from clan I and clan J. We prepared HEK 293 cells by transforming them with a plasmid that contained luciferase cDNA under the control of the NF-kB promoter, which was activated by transformation with a plasmid containing Carma 1 cDNA. To investigate the anti-inflammatory properties of the different MAMs, cells were co-transformed with MAM cDNA that was cloned in the eukaryotic expression plasmid ProbiH1. The resulting cells exhibited a wide range of anti-inflammatory activity in the NF-kB reporter luciferase assay depending on the MAM protein introduced (Figure 5). MAMs from strains M21/2, SL3/3, AHMP21, CNCM4575, and APC942/8-14-2 clan I were associated with the strongest reductions of the NF-kB pathway, with at least 85% inhibition. MAMs from strains A2-165, KLE1255, APC942/8-14-2 clan J, L2-6 clan G, and L2-6 clan H showed a moderate degree of inhibition, between 40% and 80%. Instead, the MAM from strain CNCM4541 was the only one with no detectable inhibitory activity. We also determined that MAM from M21-2 was two times more efficient than MAM from A2-165, meaning that the same levels of NF-kB inhibition were obtained with half the quantity of MAM M21-2 plasmid (data not shown). To assess the specificity of the inhibition, we tested two eukaryotic proteins, beta-lactoglobulin from cow’s milk and the IL-18 receptor, but were not able to detect any inhibition of luciferase activity for the same amount of plasmid transfected (data not shown).

We next examined in more detail whether MAMs from the same clan exhibited similar levels of anti-inflammatory activity. As shown in Figure 5, MAMs from M21/2 and SL3/3 (clan A) displayed similarly high anti-inflammatory activity. However, MAMs from strains A2-165 and AHMP21 (clan B) were associated with 43% and 11% reductions of the NF-kB pathway, respectively. Thus, despite a high degree (83%) of sequence similarity, MAMs from A2-165 and AHMP21 clearly had different capacities for anti-inflammatory activity. The anti-inflammatory activity of each MAM has been compared one to one and the results are indicated in Table S3.

We performed an immunoblotting experiment to detect the level of each MAM expression (Figure S2). All MAMs were detected except for MAM APC942_8_14_2 clan J. Surprisingly, the level pf expression was different from one MAM to another.

### 2.6. In Vivo Comparison of Anti-Inflammatory Activity of MAM from F. prausnitzii A2-165 and M21-2 in a Model of Acute Colitis

**Figure 6:**
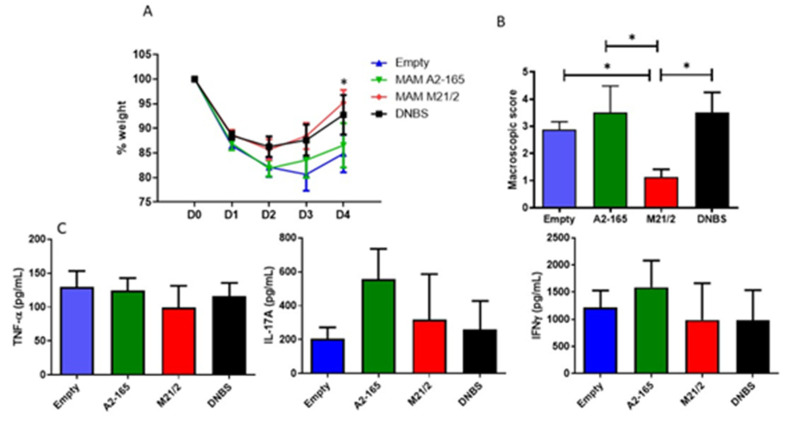
Protective effect of MAM A2-165 or MAM M21/2 in a DNBS-induced model of acute colitis. Recombinant bacteria containing vectors with MAM from strain A2-165, MAM from strain M21/2, or proBiH1-EMPTY were administered orally to mice (*n* = 8/group) for 7 days prior to colitis induction by intrarectal injection of DNBS (150 mg/kg). Mice were then sacrificed 3 days after DNBS injection. (**A**) Daily monitoring of mouse weight; (**B**) Macroscopic score; (**C**) Immune response in MLN. Data represent mean ± SD. * *p* < 0.05.

To confirm if the differences observed in vitro were relevant in vivo, we administered MAM from A2-165, MAM from M21-2, and an empty vector (Empty) to mice (*n* = 8) for 7 days and then induced colitis by intrarectal injection of DNBS. We chose to test MAM from strain A2-165 as a reference because it was the first MAM described, and MAM from strain M21/2 because it had one of the highest anti-inflammatory activities in vitro. The weight of each mouse was monitored for 4 days after colitis induction (D1-D4), and mice were sacrificed at D4. Compared to treatment with the empty vector or MAM A2-165, treatment with MAM M21-2 resulted in a significant improvement in weight recovery at D4 vs. empty treatment (Figure 6A). There was no difference between treatment with the empty vector and MAM A2-165. Macroscopic scores were also significantly lower in mice treated with MAM M21-2 compared to the groups treated with MAM A2-165 or the empty control (Figure 6B). Lymphocytes from Mesenteric Lymph nodes were retrieved, cultivated and different pro-inflammatory cytokines, TNF-α, IL17 and IFNγ, were monitored (Figure 6C). No differences between the groups were observed.

### 2.7. In Vivo Comparison of Anti-Inflammatory Activity of MAM from F. prausnitzii A2-165 and M21-2 in a Model of Chronic Colitis

**Figure 7:**
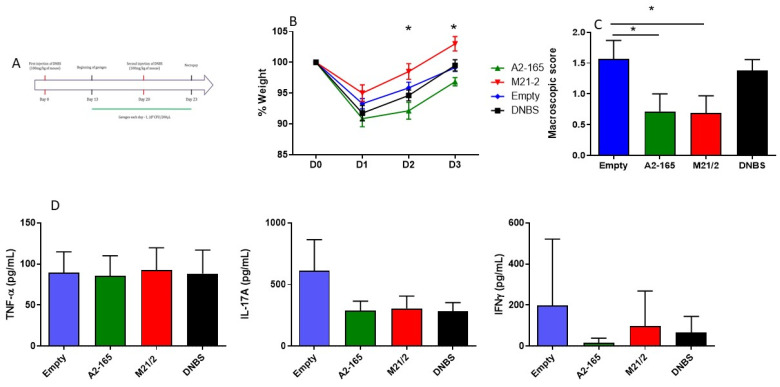
Protective effect of MAM A2-165 or MAM M21/2 in a DNBS-induced model of chronic colitis Recombinant strains were also tested in a model of chronic inflammation. Mice were administered a lower dose of DNBS (100 mg/kg) two times, with a 3-week interval between doses. (**A**) Experimental design; (**B**) Daily monitoring of mouse weight; (**C**) Macroscopic score; (**D**) Immune response in MLN. Data represent mean ± SD. * *p* < 0.05.

Both recombinant strains were also tested in a mouse model of chronic inflammation. Mice were administered a low dose of DNBS two times, with a three-week interval between doses (Figure 7A), which was designed to mimic two flares separated by three weeks of remission. The dose of DNBS used was less than in an acute model in order to induce less severe damage. In this experiment, mice treated with MAM M21-2 recovered weight more efficiently at D2 and D3 compared to mice that had been administered MAM A2-165 (Figure 7B). Macroscopic scores were better for mice treated with either MAM compared to the DNBS or Empty vector controls (Figure 7C). Lymphocytes from Mesenteric Lymph nodes were retrieved, cultivated and different pro-inflammatory cytokines, TNF-α, IL17 and IFNγ, were monitored (Figure 6C). No differences between the groups were observed.

## 3. Discussion

Since 2008, *Faecalibacterium prausnitzii* has become the subject of much research due to its anti-inflammatory properties [2] and its high abundance in the gut, where it represents up to 5% of the human microbiota [10]. Recent work has focused on elucidating the genetic diversity of this bacterium. Publications in 2012 and 2016 by Lopez-Siles et al. classified *F. prausnitzii* into two phylogroups [14,15] based on 16S rRNA sequence analysis. Subsequently, our group analyzed the phylogenetic relationships among 17 isolated strains of *F. prausnitzii* using 16S rRNA sequences as well as genomic comparative analysis, which comprised analyses of phylogenomics, whole-genome multi-locus sequence typing, average nucleotide identity, and gene synteny. Of these 17 strains, 13 clustered into three phylogenic groups (groups A, B, and C), while the remaining four strains (*F. prausnitzii* L2-6, CNCM-I-4575, AHMP_21, and CNCM-I-4541) each formed its own cluster [13]. More recently, an analysis of 31 *F. prausnitzii* genomes led the authors to propose the separation of *F. prausnitzii* into two new species, *F. prausnitzii sensu stricto* and *F. moorei* [16].

This genetic diversity seems to be accompanied by differences in functional characteristics [12]. For example, strains of *F. prausnitzii* were found to differ in their resistance to various types of antibiotics. Similarly, an investigation of the immune-modulating properties of *F. prausnitzii* strains in vitro revealed variability in their capacity to reduce IL-8 production in TNFα-induced HT-29 cells and to stimulate IL-10 production in PBMC culture. Specifically, all 13 tested strains tended to decrease IL-8 secretion, but not to the same extent. Only four strains—*F. prausnitzii* A2-165, CNCM_4543, CNCM_4573, and L2-6—were able to induce IL-10 production, with A2-165 and CNCM_4543 (both members of phylogroup B) as the most promising.

Previous work by our group identified a protein, MAM, which is produced by *F. prausnitzii* and which has anti-inflammatory properties [1]. Specifically, we were able to demonstrate that recombinant *Lactococcus lactis* that contained MAM cDNA from *F. prausnitzii* A2-165 inhibited the NF-kB pathway in a mouse model of DNBS-induced inflammation. Administration of the recombinant strain resulted in delivery of the MAM plasmid to the mouse intestinal mucosa, enabling the host to produce MAM itself. MAM produced by the host mucosa also showed the ability to reduce Th1, Th2, and Th17 immune responses in two different models of chemical-induced inflammation (DSS and DNBS). Moreover, the production of MAM resulted in increased levels of IL-10 in the DNBS model and TGFβ in the DSS model; both of these cytokines are related to the T cell regulator profile (Treg) responsible for intestinal homeostasis [8].

MAM proteins have been identified only in *Faecalibacterium* species. In the present study, comparison of the MAM-encoding gene regions in various strains of *F. prausnitzii* revealed that the current state of this region is the product of numerous rearrangement events. Interestingly, we observed the presence of neighboring tRNA genes in all strains (black blocks in Figure 1B), and in strain CNCM4540 in particular, two tRNA-Ala genes were identified as flanking the *mam* region. In bacteria, tRNA genes are known to be hotspots that exhibit frequent homologous recombination-driven diversification [22]. The organization of the *mam* regions suggests that they have been subjected to a long-term, continuous, and possibly ongoing process of recombination, which may explain why some strains have two *mam* genes. The potential of *mam* genes for rapid evolutionary change may then be the source of the functional diversification described.

In all *F. prausnitzii* genomes investigated, the *mam* gene is located near a cluster of genes potentially involved in sporulation; this is intriguing given that, to date, the ability of *F. prausnitzii* to sporulate remains an enigma. In addition, we noted (Figure 1) that in certain strains the *mam* gene was located near a PCAT with a characteristic N-terminal peptidase C39 domain. C39 cysteine proteases cleave the “double-glycine” leader peptides in export proteins. All MAM proteins share consensus sequences in the N-terminal part and a common processing site, with two conserved glycine residues in positions 20 and 21 (Figure S1). It is tempting to speculate that the neighboring PCAT is involved in the cleavage and translocation of MAM proteins across the cytoplasmic membrane to the extracellular space. This hypothesis is supported by the finding that several MAM-derived peptides lacking the N-terminal section were detected in the supernatant of strain A2-165 [1]. PCAT transporters play key roles in shaping how bacteria interact with each other. Their most common function is to assist in the biosynthesis of antimicrobial peptides (i.e., bacteriocins) produced by bacteria to kill or inhibit the proliferation of other, usually closely related, bacteria [23,24]. Some PCATs also promote cell-to-cell communication by secreting peptide pheromones in Gram-positive quorum-sensing systems [25]. The role of this PCAT transporter in processing and transporting MAMs, as well as the precise cellular localization of MAMs, requires further investigation.

To date, most research on the anti-inflammatory activities of *F. prausnitzii* in murine models of colitis has been conducted using strain A2-165 [1,2,8]. Through a combination of in vitro and in vivo approaches, this study provides the first evidence that MAM from strain M21/2 has stronger anti-inflammatory potential—i.e., NF-kB inhibition—than the corresponding protein from strain A2-165 (Figure 5 and Figure 6). We hypothesize that these dissimilarities in anti-inflammatory activity might be the result of differences in the amino acid sequences of the MAM proteins. By applying a sequence clustering approach, we were able to define at least 10 clans of MAM proteins, which overlap, at least in part, the previously defined phylogroups of *F. prausnitzii* [13]. Using an NF-kB reporter luciferase assay, we showed that MAM proteins from different strains exhibit a wide range of anti-inflammatory activity. Interestingly, the MAMs from strains A2-165 and AHMP21 share 83% sequence similarity but had starkly different inhibitory capacities (by a factor of 4). The explanation for this apparent contradiction may lie in the three-dimensional structure of MAM proteins, which to date remains unknown. Our results underline the importance of filling this gap in order to better understand the relationships between the structure of MAMs and their anti-inflammatory activity.

Finally, we compared the in vivo protective effect of MAM from A2-165 and M21/2 in two models of DNBS-induced colitis, acute and chronic. In the acute model, treatment with MAM 21/2 promoted weight recovery and reduced macroscopic scores by a factor of 3 compared to controls, while MAM A2-165 had no effect on either result. In the chronic model, both MAM A2-165 and MAM M21/2 decreased macroscopic scores by a factor of 2 compared to the DNBS control group, but only MAM M21/2 was linked with improved weight recovery. These results confirmed that, in vivo, MAMs from different strains exhibit different anti-inflammatory properties.

The mechanisms underlying the differences in anti-inflammatory activity among *F. prausnitzii* strains seem to be very complex. Many potential candidates have been suggested, including the production of butyrate [26,27], shikimic acid, and salicylic acid [28]; the modulation of Toll-like receptor 2 [6]; the induction of IL-10–secreting T cells [29]{Rossi, 2015 #4133}; and the production of IL-12 and IL-10 [6]. Further studies are needed that integrate all of these parameters with our data on the anti-inflammatory properties of MAM, which can then be used to develop a general model of the anti-inflammatory potential of the various species of *Faecalibacterium*.

## 4. Materials and Methods

### 4.1. Data Collection, Sequence and Phylogenetic Analysis

MAM-like sequences were retrieved by running Psi-BLAST searches (3 iterations, inclusion threshold E-value 0.001) against the non-redundant protein sequence database using the MAM sequence from strain A2-165 as query. The results were manually inspected and partial protein sequences were removed. The resulting MAM dataset included 108 sequences (Table S1). The MAM sequences were then clustered using Cluster Analysis of Sequences (CLANS) [21], a Java utility based on the Fruchterman-Reingold graph-layout algorithm that runs BLAST on a given set of sequences, all-against-all, and clusters them in three dimensions according to their similarities. A 2D-representation was obtained by seeding sequences randomly in the arbitrary distance space. The sequences within each cluster were aligned with ClustalW using Bioedit V7 [30]. The evolutionary history of MAMs was inferred using the maximum-likelihood method and the JTT matrix-based model [31]. Evolutionary analyses were conducted in MEGA X with 500 bootstrap replicates [32].

### 4.2. Plasmid and Strains

Synthetic cDNA (GeneCust, Boynes, France) of MAMs from different *F. prausnitzii* strains (A2-165, M21/2, SL3/3, KLE1255, CNCM 4541, CNCM4575, L2-6, and APC942_8_17_2) (Supplementary Table S1) was optimized for eukaryotic expression and inserted into the proBiH1 plasmid, which contained the repA and repC origins of replication, the chloramphenicol resistance gene, and the expression cassette from pcDNA3 (Invitrogen; Ilkirch, France). All plasmids constructs were validated by restriction enzyme analysis and sequencing. The resulting plasmids, each containing a different MAM, were cloned into *Lactococcus lactis* MG1363 to create the recombinant strains LL-proBiH1.

LL-proBiH1 strains were preincubated at 30 °C overnight in M17 broth medium with 0.5% glucose and chloramphenicol (10 µg/mL); initial culture OD was 0.2 and culturing ended at OD 1.7–1.8. Cell counts were quantified on M17 agar plates with 0.5% glucose (10 µg/mL) and chloramphenicol (10 µg/mL) to assess viable CFU concentrations. Bacterial suspensions were stored in PBS with 16% glycerol, and 10^9^ CFU of bacteria were administered orally to mice every day.

### 4.3. HEK-293 Culture Conditions, Transfected Cell Line, NF-κB Reporter Assay and MAM Immunoblotting

The human embryonic kidney cell line HEK-293 was obtained from the American Type Culture Collection (ATCC). HEK-293 cells were maintained in Dulbecco’s modified Eagle’s medium + glutaMAXTM (DMEM, Gibco, Ilkirch, France), supplemented with 10% heat-inactivated fetal bovine serum (Eurobio, Les Ulis, France), 1% sodium pyruvate (Gibco, Ilkirch, France), and 1% streptomycin/penicillin (Sigma, L’lsle-d’Abeau Chesnes, France), and grown at 37 °C in a humidified atmosphere of 10% CO_2_. Cells were passed at 80% confluence and seeded in 24-well plates at 8×10^4^ cells/well, then incubated for 24 h. Transfection was performed using Lipofectamine LTX with Plus Reagent (Invitrogen, Ilkirch, France) in opti-MEM^®^ medium (Gibco, Ilkirch, France). Different concentrations were used for the different plasmids of interest: 0.2 µg/well of the proBiH1 plasmids or Carma1 and 0.02 µg/well of the NF-kB luciferase reporter plasmid. HEK-293 cells were co-incubated with each plasmid mix for 4 h, and then the medium was replaced with a complete medium. Twenty-four hours later, reactive Luciferase Assay reagent (Promega, Charbonniéres les bains, France) was added to lysed cells in Passive Lysis Buffer 1X (Promega, Charbonniéres les bains, France) and luminescence was measured with an Infinite M200 pro device (TECAN, Neuville sur Oise, France). Each plasmid was tested in triplicates (technical replicate) and experiments were performed three times (biological replicate). Results are expressed as mean of the three biological replicates. Immunoblotting targeting the FLAG region of the MAM expressed protein was used to detect MAM produced in HEK293.

### 4.4. Animals and Experimental Design

All animal experiments were performed according to European Community rules of animal care and approved by the local Ethical Committee Comethea (authorization n° 16744-201807061805486, delivered by the French ministry of research).

Seven-week-old male C57BL/6J mice were obtained from Janvier Lab and maintained under specific pathogen-free conditions in the animal facilities of the National Research Institute for Agriculture, Food, and the Environment (IERP, INRAE). We tested the effects of treatment with lactococci carrying proBiH1 EMPTY, proBiH1 MAM A2-165, or proBiH1 MAM M21/2 in two models of colitis, acute and chronic.

Acute model: After 7 days of acclimation, mice were divided into 5 groups (*n* = 8 mice/group) and housed in cages of 4. Mice in the control vehicle and DNBS vehicle groups received a solution of 200 µL of PBS with 16% glycerol; treated mice received 5 × 10^9^ CFU of lactococci that carried either proBiH1 EMPTY, proBiH1 MAM A2-165, or proBiH1 MAM M21/2, in a solution of 200 µL PBS with 16% glycerol. All solutions were administered orally. One week after the start of daily force-feeding, mice were anesthetized by intraperitoneal injection of 0.1% ketamine and 0.06% xylazine and received by rectal injection a solution of 150 mg/kg of dinitrobenzene sulfate (DNBS) dissolved in 30% ethanol. The control vehicle group (no inflammation) received the same intrarectal injection with a solution of 30% ethanol only. Three days after DNBS injection, mice were euthanized. Body weight variation was measured daily, and Wallace’s scores of macroscopic damage (Wallace et al., 1989), with some modifications, were determined as follows. Tissue sections from each mouse were scored by evaluating ulcerations (score of 0–5); adhesions (presence/absence: 0/1); hyperemia (presence/absence: 0/1); altered transit, such as diarrhea or constipation (presence/absence: 0/1); and increases in colon wall thickness (presence/absence: 0/1; measured using an electronic caliper, Control Company, WVR, United States).

Chronic model (Figure 7A): After 7 days of acclimation, mice were divided into 5 groups (*n* = 8 mice/group)—control vehicle, DNBS vehicle, LL-proBiH1 EMPTY, LL-proBiH1 MAM A2-165, and LL-proBiH1 MAM M21/2—and housed in cages of 4. Mice were anesthetized by intraperitoneal injection of 0.1% ketamine and 0.06% xylazine and received by rectal injection a solution of 100 mg/kg of DNBS dissolved in 30% ethanol. The control vehicle group (no inflammation) received the same intrarectal injection with a solution of 30% ethanol only. Thirteen days after DNBS injection (D13), control vehicle and DNBS vehicle mice received a solution of 200 µL of PBS with 16% glycerol; treated mice received 5x10^9^ CFU of lactococci that carried either proBiH1 EMPTY, proBiH1 MAM A2-165, or proBiH1 MAM M21/2 in a solution of 200 µL PBS with 16% glycerol. All solutions were administered orally. Seven days after the beginning of treatment (D20), mice were anesthetized by intraperitoneal injection of 0.1% ketamine and 0.06% xylazine and received by rectal injection a second dose of 100 mg/kg of DNBS dissolved in 30% ethanol. The control vehicle group received the same intrarectal injection with a solution of 30% ethanol only. Three days after DNBS injection (D23), mice were euthanized. Body weight variation was measured daily and macroscopic scores were determined as described above.

### 4.5. Lymphocyte Isolation and Measurement of Cytokine Production

Lymphocyte suspensions were prepared from the Mesenteric Lymph Nodes (MLN) by pressing cells through a 70-mmol/L Falcon nylon cell strainer (BD Biosciences, San Jose, CA, USA). Lymphocytes were counted by flow cytometry (Accuri C6) and resuspended in culture medium (RPMI, Lonza with 100 units of Streptomicin Penicilin, PAA Laboratories and 10% SVF, Lonza) and activated with coated anti-mouse antibody CD3e and CD28 (eBioscience, Ilkirch, France). Cytokine production was assessed in supernatant by ELISA (TNFα, IL-17A and INF-γ, Mabtech, Nacka Strand, Sweden) after 48 h of incubation.

### 4.6. Statistical Analysis

Graphics were created in Prism-GraphPad^®^. Data are represented as mean ± standard deviation (SD) and were analyzed by Kruskal–Wallis test or analysis of variance (ANOVA) after tests for normalization. In all results, * *p* < 0.05, ** *p* < 0.01, and *** *p* < 0.001.

## Data Availability

Not applicable.

## Supplementary Materials

The following supporting information can be downloaded at: https://www.mdpi.com/article/10.3390/ijms23031705/s1.
