# Supplementary material for: Intraspecific Diversity of Microbial Anti-Inflammatory Molecule (MAM) from Faecalibacterium prausnitzii
Source: Int J Mol Sci. 2022 Feb 1;23(3):1705. doi: 10.3390/ijms23031705 (PMC8836110; doi:10.3390/ijms23031705)

Figure S1. ClustalW Multiple Alignment of MAM sequences within each clan.

Clan A (red)

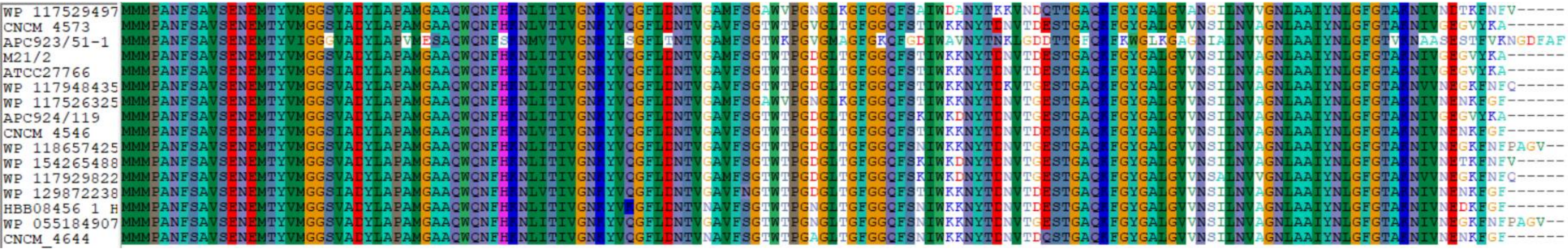

Clan B (green)

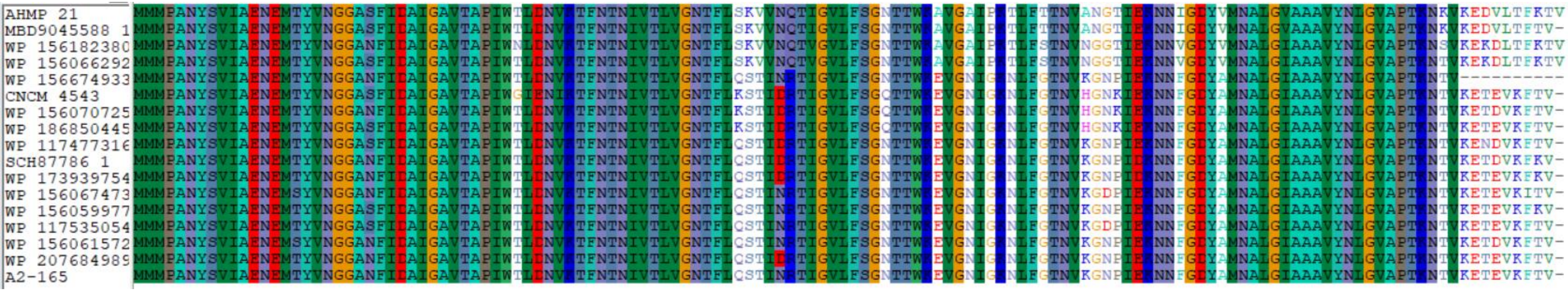

Clan C (blue)

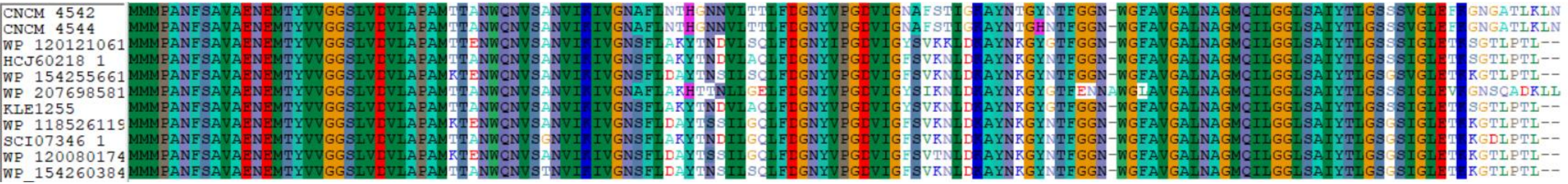

Clan E (black)

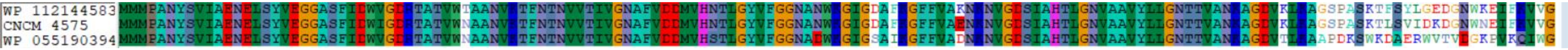

Clan G (light blue)

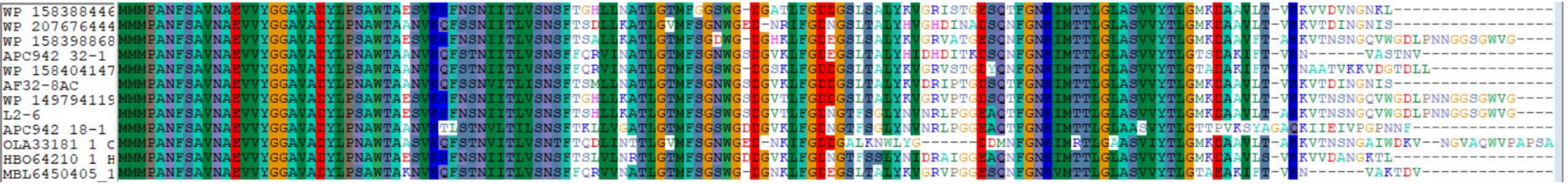

Clan H (pink)

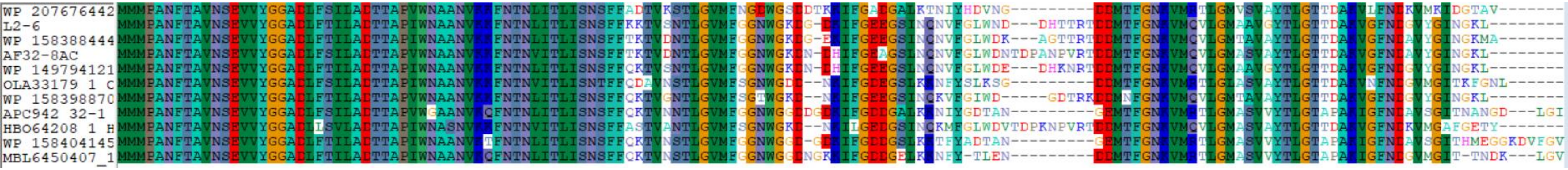

Clan I (yellow)

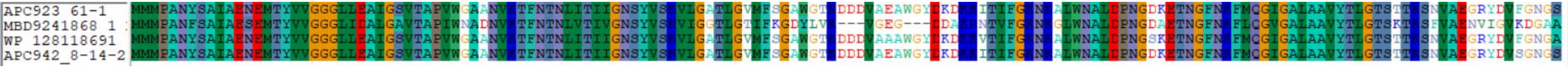

Clan J (purple)

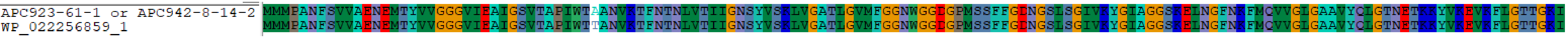

Supplement: Supplementary file 1 [file ijms-23-01705-s001.zip › ijms-1420032 - Sypplementary revised/Figure S1.pdf]
