# Supplementary figures and images for: Intraspecific Diversity of Microbial Anti-Inflammatory Molecule (MAM) from Faecalibacterium prausnitzii
Source: Int J Mol Sci. 2022 Feb 1;23(3):1705. doi: 10.3390/ijms23031705 (PMC8836110; doi:10.3390/ijms23031705)

Figure S2. Immunoblot analysis of MAM expression in HEK 293

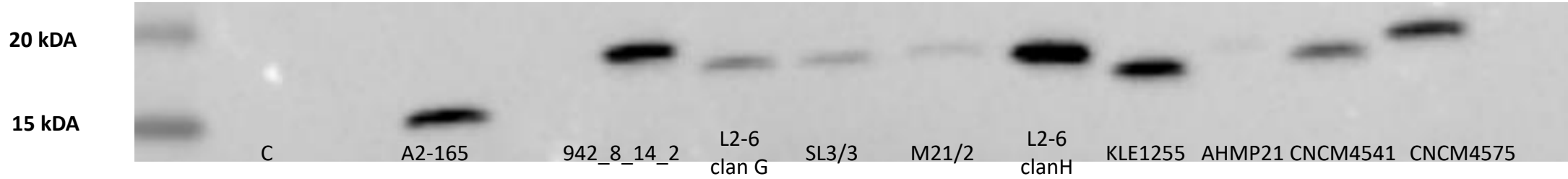

Supplement: Supplementary file 1 [file ijms-23-01705-s001.zip › ijms-1420032 - Sypplementary revised/Figure S2.pdf]
